# Supplementary material for: An Optimized LIVE/DEAD Assay Using Flow Cytometry to Quantify Post-Stress and Antifungal-Treatment Survival in Diverse Yeasts
Source: bioRxiv. 2025 Jun 2:2025.04.14.648826. Preprint. [Version 2] doi: 10.1101/2025.04.14.648826 (PMC12157429; doi:10.1101/2025.04.14.648826)
Supplement: Supplement 1 [file NIHPP2025.04.14.648826v2-supplement-1.pdf]

Supporting Information Captions

Fig. S1 Viability of mock-treated mid-log phase *C. glabrata* cells. Ratios between the CFU and the number of cells plated based on flow cytometry in mock-treated (mid-log) *C. glabrata* cells resuspended in the indicated buffers. Dots represent the individual biological replicate and bars represent the mean.

Fig. S2 Testing dye concentrations, incubation time and dye ratio on the staining

bioRxiv preprint doi: https://doi.org/10.1101/2025.04.14.648826; this version posted June 2, 2025. The copyright holder for this preprint (which was not certified by peer review) is the author/funder, who has granted bioRxiv a license to display the preprint in perpetuity. It is made available under aCC-BY 4.0 International license.

pattern. (A) 2D density plots showing staining patterns of mid-log *C. glabrata* cells at various dilutions of SYTO 9 or PI (stock concentrations: 3.34 mM for SYTO 9, 20 mM for PI). Rectangular gates were used to quantify cell events considered damaged or dead. (B) Median Fluorescence Intensity (MFI) over time for SYTO 9 stained mid-log *C. glabrata* cells. Dots show individual biological replicates (n = 4, biological replicates). Thick red bars represent the mean and the error bars represent the 95% confidence intervals. (C) Similar to B for PI-stained, heat-killed *C. glabrata* cells. (D) 2D density plots for *C. glabrata* cells treated with H_2O_2 and stained with various ratios of PI:SYTO 9 (right). For all samples, SYTO 9 is applied at a fixed concentration of 3.34 μM; PI is used at 20 μM at 1:1 ratio.

Fig. S3 H_2O_2 treatment causes changes in cell size as indicated by FSC (forward scattering). FSC.H (height) distribution of gated single cell events were shown for each of the three species as indicated on the top. Cells were treated for 2 hours at four different H_2O_2 doses (y-axis labels). Three biological replicates were shown for each species and treatment.

Fig. S4 Normalizing fluorescence by FSC.H didn't reduce the sample variance dramatically.. Fluorescence signals were first normalized by FSC.H (see Methods for details) and then the same gating strategies were applied as for the unnormalized data to calculate the percent live values. The resulting estimates were plotted side-by-side with the non-normalized (Fig. 4B) results for *C. glabrata* treated with different concentrations of H_2O_2. Statistical test results were shown below the graph similarly to

bioRxiv preprint doi: https://doi.org/10.1101/2025.04.14.648826; this version posted June 2, 2025. The copyright holder for this preprint (which was not certified by peer review) is the author/funder, who has granted bioRxiv a license to display the preprint in perpetuity. It is made available under aCC-BY 4.0 International license.

Fig. 4B.

Fig. S5 Example gating strategy for flow cytometry data. (A) All events were plotted on FSC.H and SSC.H. A rectangular gate is used to exclude non-cell events. (B) Events within the first gate (“cell”) were plotted on FSC.H and FSC.W. Singlets (single cell event as opposed to doublets or multilets) were selected by excluding events with a higher FSC.W given the same FSC.H.

bioRxiv preprint doi: https://doi.org/10.1101/2025.04.14.648826; this version posted June 2, 2025. The copyright holder for this preprint (which was not certified by peer review) is the author/funder, who has granted bioRxiv a license to display the preprint in perpetuity. It is made available under aCC-BY 4.0 International license.

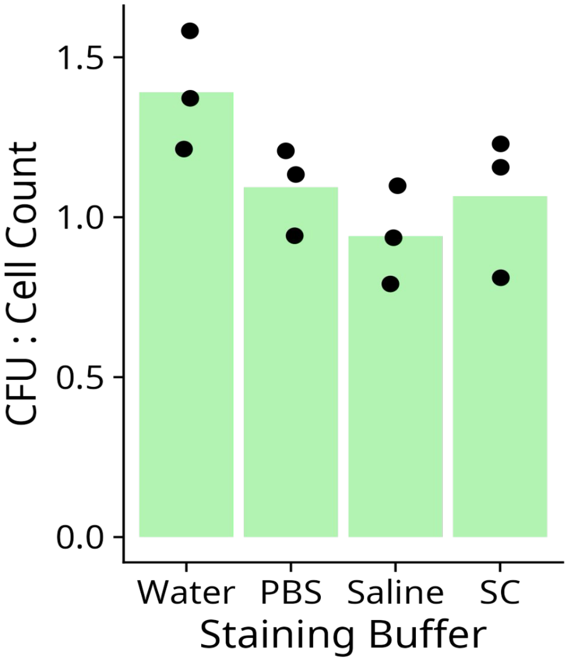

Fig. S1 Viability of mock-treated mid-log phase *C. glabrata* cells. Ratios between the CFU and the number of cells plated based on flow cytometry in mock-treated (mid-log) *C. glabrata* cells resuspended in the indicated buffers. Dots represent the individual biological replicate and bars represent the mean.

bioRxiv preprint doi: https://doi.org/10.1101/2025.04.14.648826; this version posted June 2, 2025. The copyright holder for this preprint (which was not certified by peer review) is the author/funder, who has granted bioRxiv a license to display the preprint in perpetuity. It is made available under aCC-BY 4.0 International license.

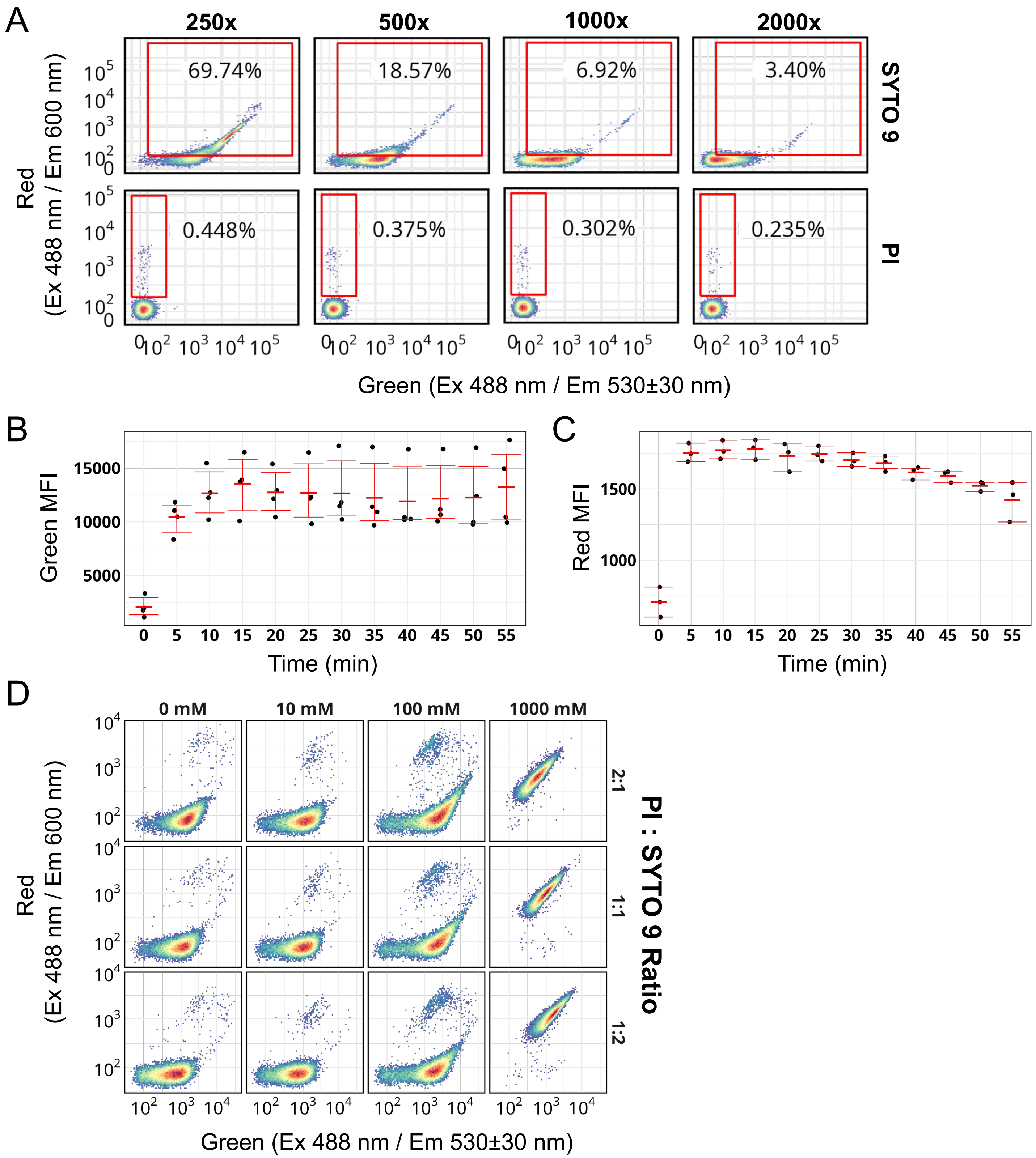

Fig. S2 Testing dye concentrations, incubation time and dye ratio on the staining pattern. (A) 2D density plots showing staining patterns of mid-log *C. glabrata* cells at various dilutions of SYTO 9 or PI (stock concentrations: 3.34 mM for SYTO 9, 20 mM for PI). Rectangular gates were used to quantify cell events considered damaged or dead. (B) Median Fluorescence Intensity (MFI) over time for SYTO9- stained mid-log *C. glabrata* cells. Dots show individual biological replicates (n = 4, biological replicates). Thick red bars represent the mean and the error bars represent the 95% confidence intervals. (C) Similar to B for PI-stained, heat-killed *C. glabrata* cells. (D) 2D density plots for *C. glabrata* cells treated with H_2O_2 and stained with various ratios of PI:SYTO 9 (right). For all samples, SYTO 9 is applied at a fixed concentration of 3.34 μM; PI is used at 20 μM at 1:1 ratio.

bioRxiv preprint doi: https://doi.org/10.1101/2025.04.14.648826; this version posted June 2, 2025. The copyright holder for this preprint (which was not certified by peer review) is the author/funder, who has granted bioRxiv a license to display the preprint in perpetuity. It is made available under aCC-BY 4.0 International license.

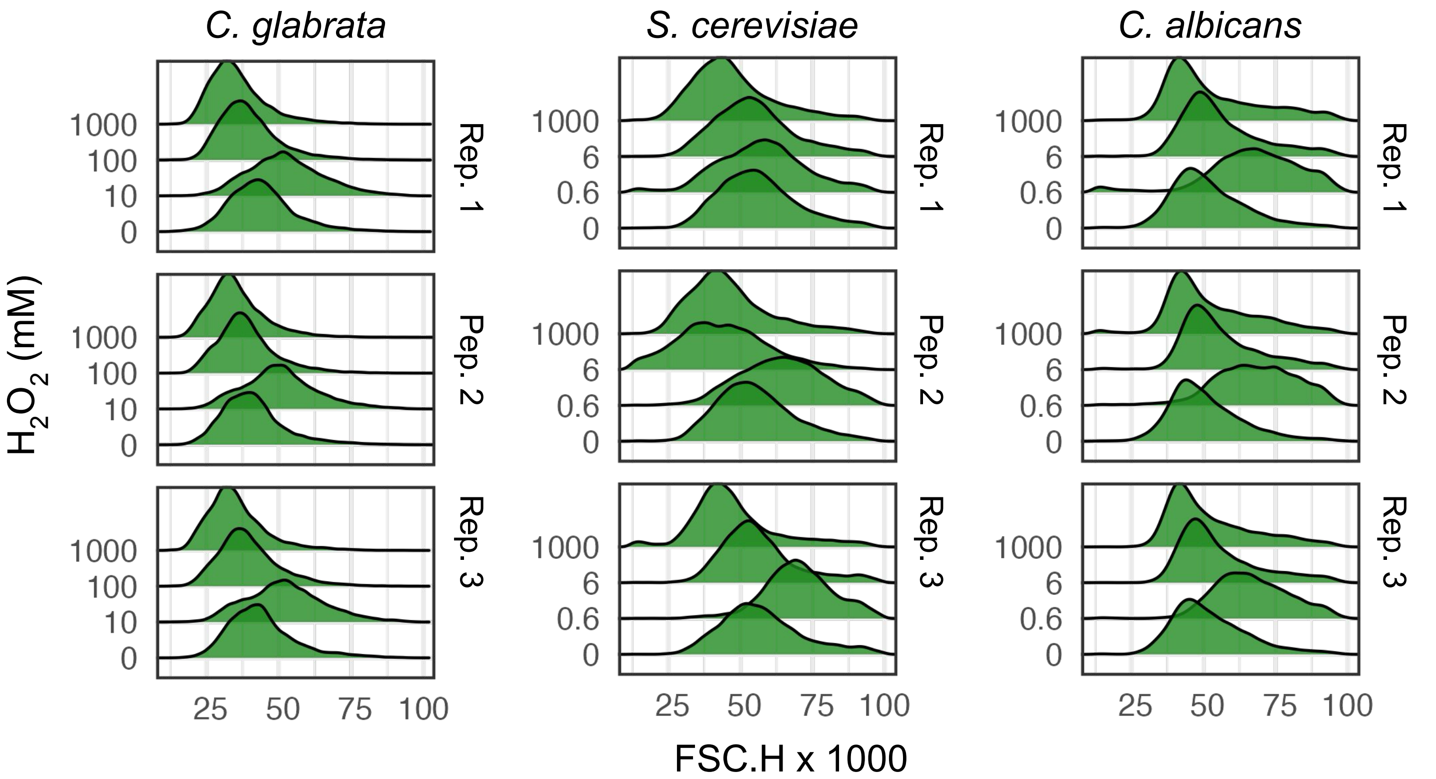

Fig. S3 H_2O_2 treatment causes changes in cell size as indicated by FSC (forward scattering). FSC.H (height) distribution of gated single cell events were shown for each of the three species as indicated on the top. Cells were treated for 2 hours at four different H_2O_2 doses (y-axis labels). Three biological replicates were shown for each species and treatment.

bioRxiv preprint doi: https://doi.org/10.1101/2025.04.14.648826; this version posted June 2, 2025. The copyright holder for this preprint (which was not certified by peer review) is the author/funder, who has granted bioRxiv a license to display the preprint in perpetuity. It is made available under aCC-BY 4.0 International license.

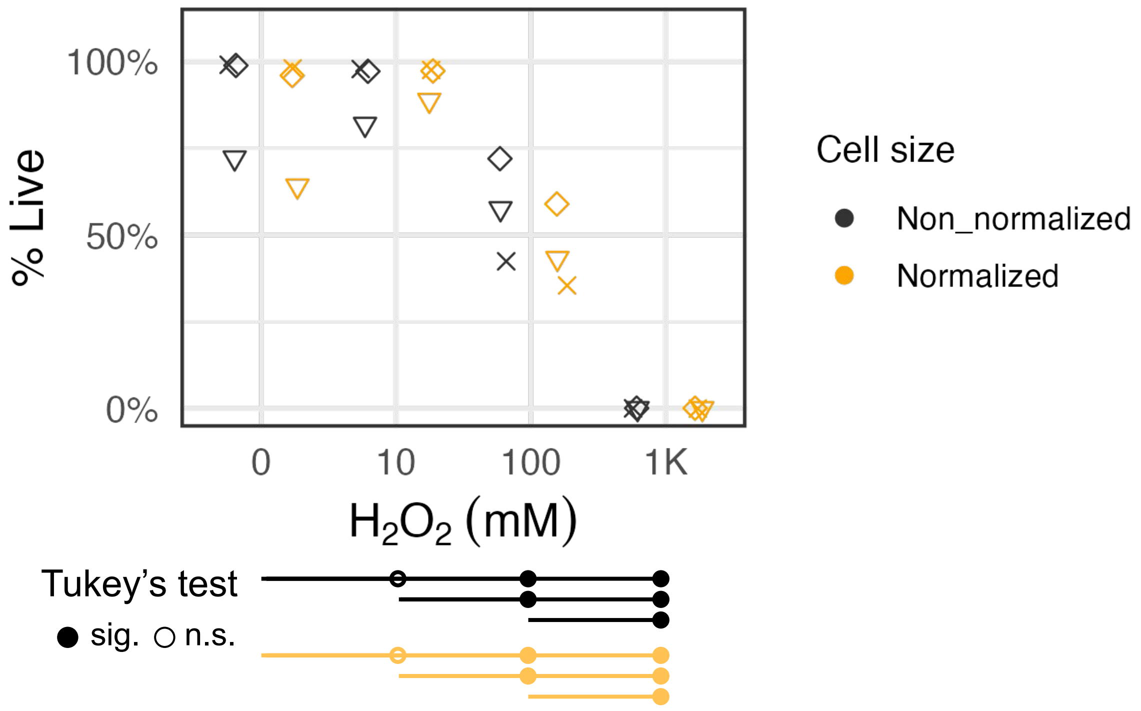

Fig. S4 Normalizing fluorescence by FSC.H didn't reduce the sample variance dramatically.. Fluorescence signals were first normalized by FSC.H (see Methods for details) and then the same gating strategies were applied as for the unnormalized data to calculate the percent live values. The resulting estimates were plotted side-by-side with the non-normalized (Fig. 4B) results for *C. glabrata* treated with different concentrations of H_2O_2. Statistical test results were shown below the graph similarly to Fig. 4B.

bioRxiv preprint doi: https://doi.org/10.1101/2025.04.14.648826; this version posted June 2, 2025. The copyright holder for this preprint (which was not certified by peer review) is the author/funder, who has granted bioRxiv a license to display the preprint in perpetuity. It is made available under aCC-BY 4.0 International license.

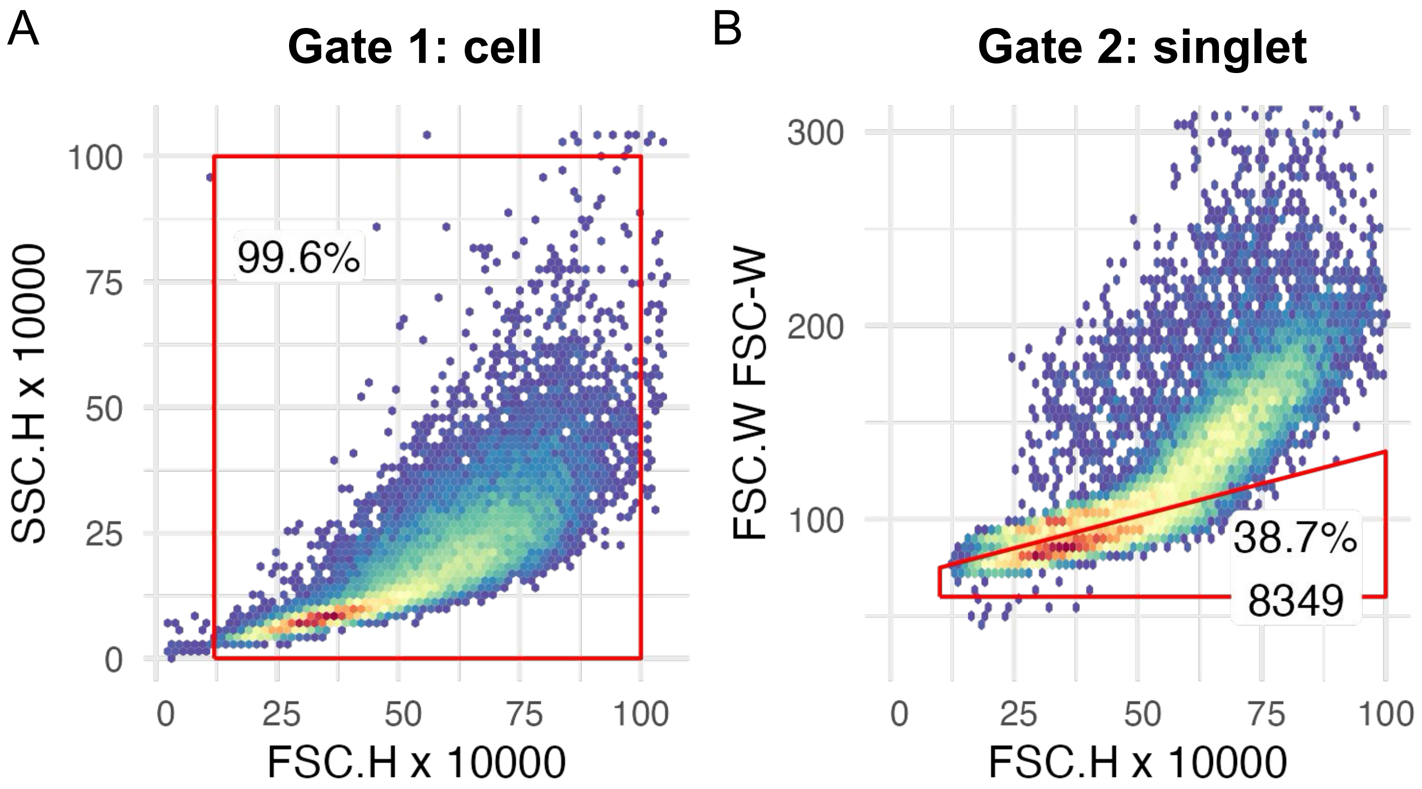

Fig. S5 Example gating strategy for flow cytometry data. (A) All events were plotted on FSC.H and SSC.H. A rectangular gate is used to exclude non-cell events. (B) Events within the first gate ("cell") were plotted on FSC.H and FSC.W. Singlets (single cell event as opposed to doublets or multilets) were selected by excluding events with a higher FSC.W given the same FSC.H.
